# Supplementary figures and images for: CAZymes in Maribacter dokdonensis 62–1 From the Patagonian Shelf: Genomics and Physiology Compared to Related Flavobacteria and a Co-occurring Alteromonas Strain
Source: Front Microbiol. 2021 Apr 12;12:628055. doi: 10.3389/fmicb.2021.628055 (PMC8072126; doi:10.3389/fmicb.2021.628055)

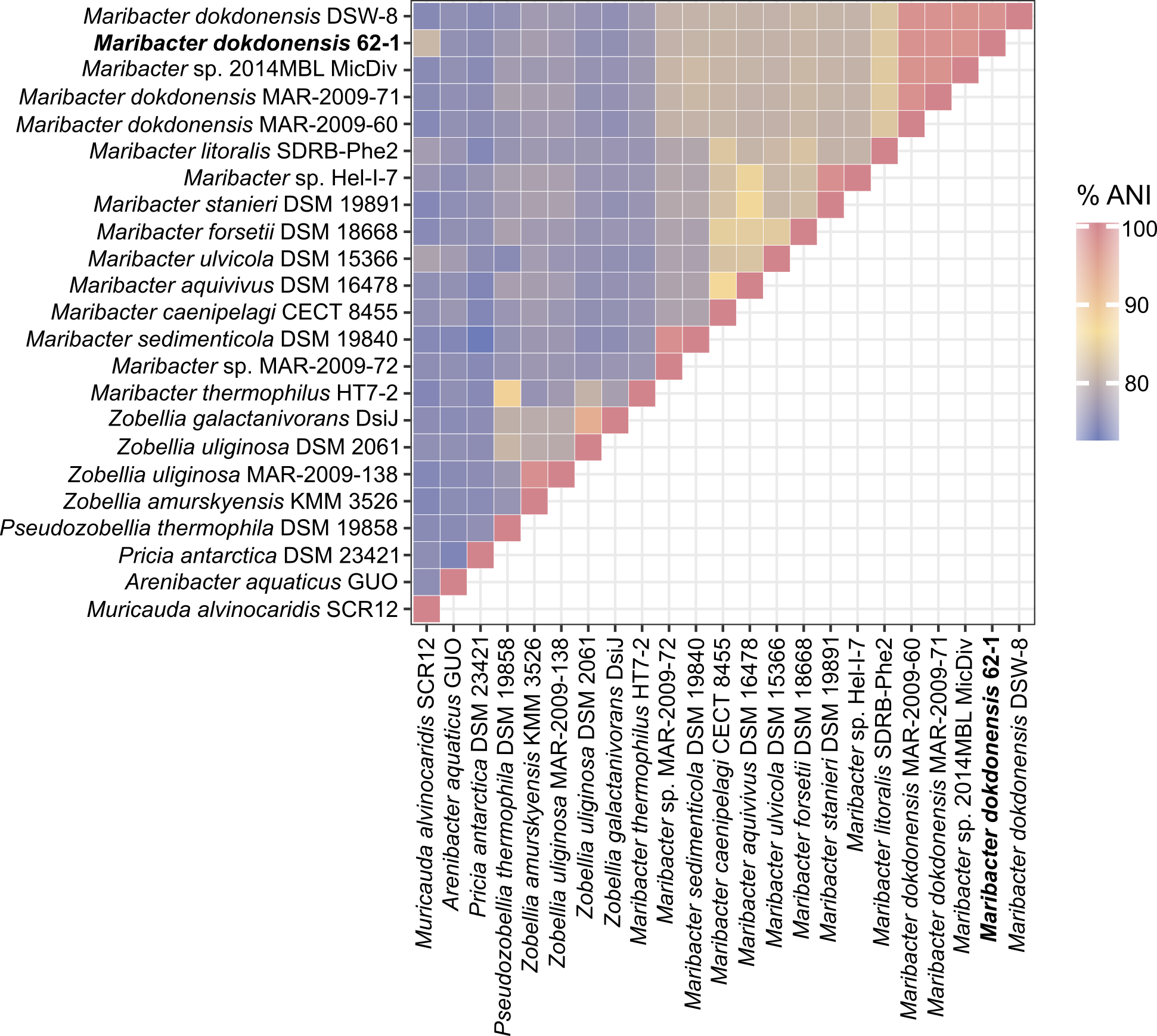

Supplement: Supplementary Figure 1 — Heatmap of average nucleotide identities (ANI) between the genomes of Maribacter dokdonensis 62–1 and related strains. [file Image_1.png]

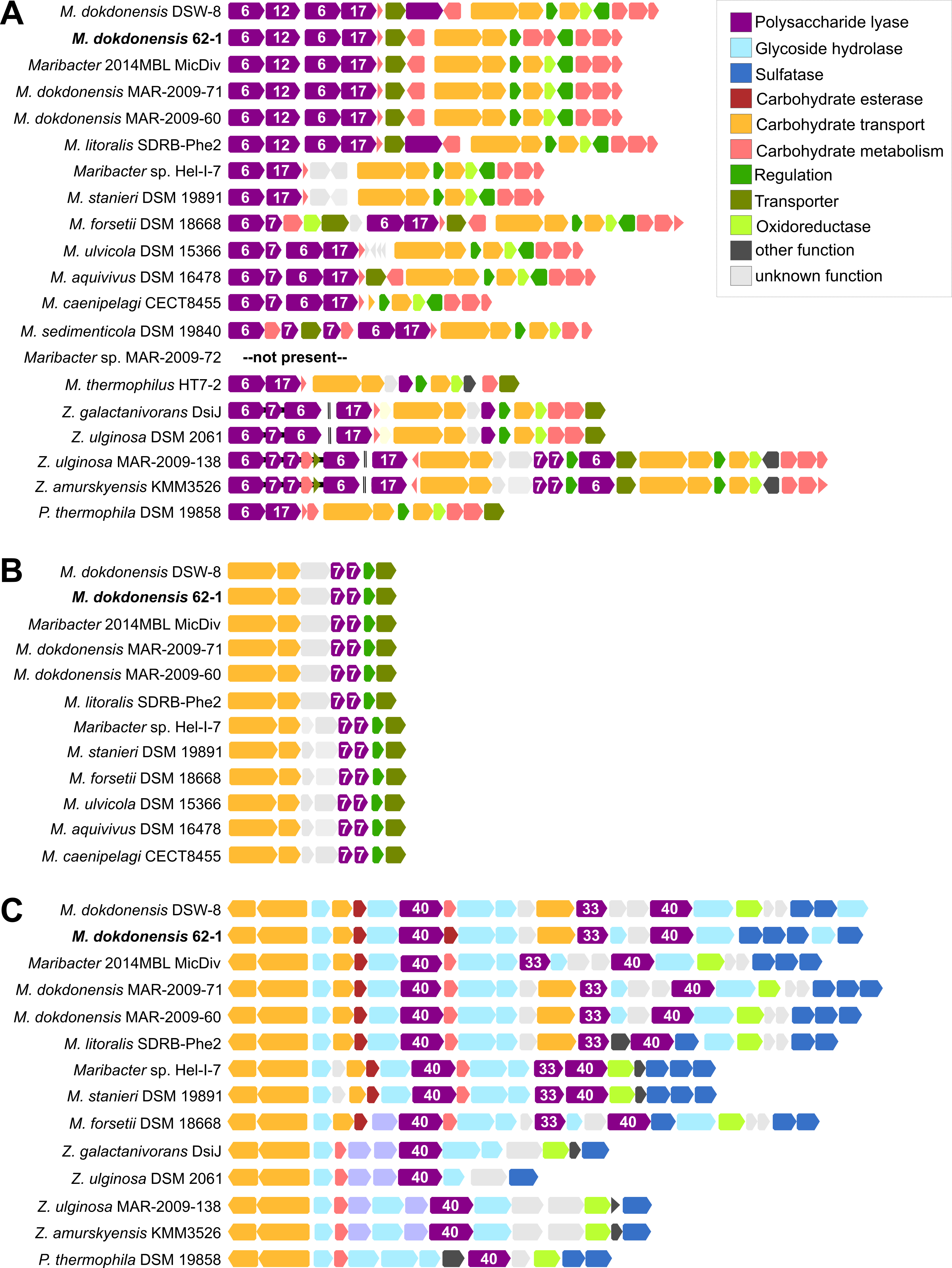

Supplement: Supplementary Figure 2 — Homologs of AlgPUL1 (A), AlgPUL2 (B) and the mixed-feature PUL relating to ulvan and fucoidan (C) in genomes of Maribacter and Zobellia strains. Numbers designate PL families. [file Image_2.png]
